# Prenatal exposure to ambient air pollution is associated with neurodevelopmental outcomes at 2 years of age

**DOI:** 10.1186/s12940-022-00951-y

**Published:** 2023-01-24

**Authors:** Zachariah E. M. Morgan, Maximilian J. Bailey, Diana I. Trifonova, Noopur C. Naik, William B. Patterson, Frederick W. Lurmann, Howard H. Chang, Bradley S. Peterson, Michael I. Goran, Tanya L. Alderete

**Affiliations:** 1 Department of Integrative Physiology, University of Colorado Boulder, Boulder, CO USA; 2 Sonoma Technology, Inc, Petaluma, CA USA; 3 Rollins School of Public Health, Emory University, Atlanta, GA USA; 4 Department of Pediatrics, The Saban Research Institute, Children’s Hospital Los Angeles, University of Southern California, Los Angeles, CA USA

**Keywords:** Neurodevelopment, Air Pollution, Health Disparities, Child Development, Pregnancy Exposures

## Abstract

**Background:**

Higher prenatal ambient air pollution exposure has been associated with impaired neurodevelopment in preschoolers and school-aged children. The purpose of this study was to explore the relationships between prenatal ambient air pollution exposure and neurodevelopment during infancy.

**Methods:**

This study examined 161 Latino mother-infant pairs from the Southern California Mother’s Milk Study. Exposure assessments included prenatal nitrogen dioxide (NO_2_) and particulate matter smaller than 2.5 and 10 microns in diameter (PM_2.5_ and PM_10_, respectively). The pregnancy period was also examined as three windows, early, mid, and late, which describe the first, middle, and last three months of pregnancy. Infant neurodevelopmental outcomes at 2 years of age were measured using the Bayley-III Scales of Infant and Toddler Development. Multivariable linear models and distributed lag linear models (DLM) were used to examine relationships between prenatal exposures and neurodevelopmental scores, adjusting for socioeconomic status, breastfeeding frequency, time of delivery, pre-pregnancy body mass index, and infant birthweight and sex.

**Results:**

Higher prenatal exposure to PM_10_ and PM_2.5_ was negatively associated with composite cognitive score (β = -2.01 [-3.89, -0.13] and β = -1.97 [-3.83, -0.10], respectively). In addition, higher average prenatal exposure to PM_10_ was negatively associated with composite motor (β = -2.35 [-3.95, -0.74]), scaled motor (β = -0.77 [-1.30, -0.24]), gross motor (β = -0.37 [-0.70, -0.04]), fine motor (β = -0.40 [-0.71, -0.09]), composite language (β = -1.87 [-3.52, -0.22]), scaled language (β = -0.61 [-1.18, -0.05]) and expressive communication scaled scores (β = -0.36 [-0.66, -0.05]). DLMs showed that higher prenatal air pollution exposure during mid and late pregnancy was inversely associated with motor, cognitive, and communication language scores.

**Conclusions:**

Higher exposure to air pollutants during pregnancy, particularly in the mid and late prenatal periods, was inversely associated with scaled and composite motor, cognitive, and language scores at 2 years. These results indicate that prenatal ambient air pollution may negatively impact neurodevelopment in early life.

**Supplementary Information:**

The online version contains supplementary material available at 10.1186/s12940-022-00951-y.

## Background

The World Health Organization estimates that more than 90% of the world’s population is exposed to particulate matter (PM) exceeding its recommended levels for healthy air and that air pollution is responsible for 7 million premature deaths annually [1]. The burden of exposure to air pollutants is unequally distributed in the US, where racial/ethnic minorities and low-income populations experience higher average levels of pollution than non-minority and high-income populations [2, 3]. For example, Latinos experience 63% more exposure to air pollution than they are responsible for creating [4]. The health consequences of these disparities are important, as epidemiological evidence suggests that polluted air is one of the leading factors associated with development of respiratory illness, cardiovascular disease, and lung cancer [5]. In addition, a growing body of evidence suggests that exposure to air pollutants has adverse influences on cognitive development in children [6, 7]. Children in general are exposed to higher levels of air pollutants due to an increased respiratory rate relative to adults [8]. Further, the emergence of cognitive capacities during childhood depends on earlier brain maturation during critical windows of development, particularly during fetal development [9]. Exposure to air pollutants during these critical periods may be more likely to adversely influence brain and cognitive development.

Studies assessing the association of prenatal air pollution exposure and cognitive development in children have predominantly focused on school aged (6–12 years) children [10–16] with most exceptions assessing these associations from early childhood (1–4 years) to school age [17–19]. Among school-aged children, prenatal exposure to polycyclic aromatic hydrocarbons (PAH) has been linked with reductions in left hemisphere white matter surface at age 8, and prenatal PM_2.5_ exposure has been shown to be inversely associated with cortex thickness, altered white matter organization, and reduced blood flow at 6–14 years. [12, 13, 20] Prenatal PM_10_ exposure has also been inversely associated with full-scale IQ at ages 4–6 years, and prenatal NO_2_ exposure and traffic density around the home has been inversely associated with verbal IQ scores at 7 years of age [10, 11]. Further, studies have shown that prenatal PM_2.5_ exposure is inversely associated with conflict network performance at ages 8–9 years, and prenatal NO_2_ exposure is associated with poorer attentional performance at 4–5 years of age [14, 15]. Additionally, studies have found that PM_2.5_ exposure between weeks 12–40 is associated with lower IQ, slower reaction times, and poorer memory at 6–7 years, [16] while NO_2_ exposure between weeks 6–14 and 32–35 is associated with increased hit reaction time at age 7 [21]. Studies assessing children younger than school age report that prenatal exposure to NO_2_ is associated with poorer cognitive development at ~14 months [19] and poorer global psychomotor development [17] between 1–6 years while prenatal PAH exposure is associated with poorer cognitive development at age 3 [18]. In addition, PM_10_, PM_2.5_, and NO_2_ exposure in the late-prenatal and early postnatal periods have been associated with an increased risk of ADHD-like behaviors in children around age 3 [22].

Previous work highlights that prenatal exposure to air pollutants is critically important since cognitive and motor capacities during childhood and beyond depend upon the proper sequencing of complex maturational events during gestation, which air pollution may disrupt, including neurogenesis, neuronal migration, axonal and dendritic arborization, synaptogenesis, myelination, apoptosis, and neural circuit formation [23–26]. Many of these processes occur throughout gestation where neurogenesis can begin as early as week six and end by mid-gestation while others, such as circuit formation and myelination, are not observed until mid-gestation and continue beyond birth at rapid pace [23–26]. Additionally, a substantial body of work highlights how disruptive events (environmental, immunological, stress) during pregnancy negatively impact neurodevelopment [27–29], where air pollution research has primarily implicated the mid-late gestational periods, when migration, synaptogenesis, myelination, and circuit formation begin to occur [24, 26, 29, 30], as critical windows of exposure [16, 21, 22].

The primary aim of the current study was to assess the association of residential prenatal exposure to ambient air pollutants—PM_2.5_, PM_10_, NO_2_—and neurodevelopmental outcomes at 2 years of age in infants from the Southern California Mother’s Milk Study. We also sought to determine whether these pollutants exert their adverse influences during critical windows of prenatal development. We hypothesized that residential exposure to ambient air pollutants during pregnancy would be associated with poorer cognitive and motor development at 2 years of age**.** We also hypothesized that the adverse outcomes associated with exposure would be most evident with higher exposure levels in late pregnancy, the time of rapid neural circuit formation.

## Methods

Participants were recruited from the Mother’s Milk Study, a longitudinal cohort of Latino mother infant pairs from Southern California [31–36]. Recruitment began in 2016 from maternity clinics associated with the University of Southern California and Children’s Hospital Los Angeles. The Mother’s Milk study was designed to examine the effects of human breast milk components on the growth and development of infants. Inclusion criteria included: 1) mothers and fathers who self-identify as Hispanic/Latino, and their infants; 2) singleton birth; 3) mother’s declared intent to breastfeed for at least 3-months; 4) mother’s enrollment within one month of the infant’s birth; 5) mother’s ability to read and comprehend English or Spanish at a 5^th^ grade level to understand study procedures and provide informed consent. Exclusion criteria included: 1) diagnosis of significant illness (including type 1 or type 2 diabetes) or eating disorder; 2) cognitive or physical constraints that prevent participation; 3) medication use that could affect physical health, metabolism, or weight; 4) current smoking (defined as more than 1 cigarette in the past week) or use of other recreational drugs; 5) pre-term birth or diagnosis of fetal abnormalities; and 6) mothers younger than 18 years of age. Written informed consent was obtained from mothers prior to enrollment. The institutional review boards of the University of Southern California, Children’s Hospital Los Angeles, and University of Colorado Boulder each gave their approval for the study.

### Clinical assessments

At the time of the current analysis, 219 participants were enrolled in the study, of whom 196 had complete residential address histories for air pollution exposure estimates during pregnancy. Of these, 166 completed the 2-year follow up visit, when neurodevelopmental outcomes were assessed in the infants. Of those with outcome data, 5 were removed due to lacking data for time of delivery, leaving us with 161 mother-infant pairs for analysis. Maternal and family medical histories, including relevant covariate data, were collected at the 1-month postpartum visit. Infant outcomes were assessed at the 2-year study visit. Maternal pre-pregnancy body mass index (BMI) (kg/m^2^) was measured by self-reported recall of pre-pregnancy height (m) and weight (kg). Infant birthweight (kg) was obtained from hospital records. Categorical time of delivery was self-reported by mothers to estimate gestational age. Values included on-time, early (≥ 2 weeks before due date), and late (≥ 2 weeks after due date). Infant breast feedings per day were based on questionnaire data with answer choices of 0–1, 1, 2, 3, 4, 5, 6, 7, and ≥ 8 breast feedings per day. We then assigned 0–1 as 0 feedings per day, 1–7 as their reported values, and ≥ 8 as 8 before treating breast feeding as a continuous variable. Socioeconomic status (SES) was calculated using a modified version of the Hollingshead Index, with missing values replaced by the median [37]. The Hollingshead index combines data on marital status, sex, educational attainment, and occupational prestige to create a numerical index of an individual’s SES [38].

### Neurodevelopmental outcomes

Neurodevelopmental outcomes were assessed at the 2-year follow up visit using the Bayley Scales of Infant and Toddler Development – Third Edition (BSID-III). Trained research personnel administered the BSID-III under the close supervision of an expert in child developmental assessment. The BSID-III provides information on 5 domains of infant development: cognitive, motor, language, social emotional, and adaptive behavior. Cognitive, motor, and language domains were assessed in an interactive examination lasting approximately 2 h. Social emotional and adaptive behavior were assessed via parent-reported questionnaires. We elected to restrict our analysis to cognitive, motor, and language domains which each produce a scaled and composite score. In addition, the motor domain includes scaled sub-scores for fine and gross motor and the language domain includes scaled sub-scores for expressive and receptive communication. Composite scores are used to describe overall development in the relative domain of the BSID-III. Raw scores for each domain are age-scaled and then transformed to scaled and composite scores that have means of 10 and 100 and standard deviations of 3 and 15, respectively. Scores are all age-adjusted.

### Ambient air pollution exposure

Participants provided residential address histories that included the entire pregnancy period. Residential addresses were geocoded using the Texas A&M Geocoder (http://geoservices.tamu.edu/Services/Geocode/). Individual residential estimates for ambient air pollutants were modeled from geocoded address data and data from the US Environmental Protection Agency’s Air Quality System (AQS) via an inverse distance-squared weighting algorithm. These estimates use data from up to four AQS stations within 50 km of participants homes and have been previously shown to exhibit reasonable error and low bias in California [39]. These pollutants include nitrogen dioxide (NO_2_) and particulate matter less than 10 and 2.5 microns in aerodynamic diameter (PM_10_ and PM_2.5_, respectively). NO_2_ was measured in parts per billion (ppb) while PM_10_ and PM_2.5_ were measured in micrograms per meter cubed (μg/m^3^). Pollution estimates are recorded hourly and daily and were then averaged across the prenatal period. We first examined the entire prenatal (pregnancy) period, estimated as the average exposure during the 9 months prior to birth since information regarding timing of conception was unavailable. We then looked at each individual monthly lag of pregnancy exposure to search for critical windows. We refer to the pregnancy period in terms of three windows, early, mid, and late, which describe the first, middle, and last three months of pregnancy, respectively.

### Statistical analysis

Two approaches were used to examine the associations of prenatal ambient pollution with developmental outcomes at 2 years. First, multivariable linear regression was used to examine the associations of prenatal air pollution exposure (9-month pregnancy period) with neurodevelopmental outcomes. Effect estimates for these analyses are reported for a one standard deviation (SD) difference in exposure. Second, distributed lag linear models (DLMs) were used to assess the associations of monthly ambient air pollution exposures during the 9 months prior to birth with developmental outcomes at 2 years. Briefly, DLMs allow for the analysis of correlated time-varying exposure variables that can have differential effects over time rather than all at once. DLMs fit a regression that simultaneously includes all monthly exposures, such that the association for a specific month is adjusted for all other exposure windows. Effect estimates for the DLMs were scaled to an interquartile range (IQR) increase across all monthly exposures for each pollutant. IQR was used in place of SD for DLMs to aid in the comparison of effect estimates between each monthly exposure window since standard deviations varied by month. Effect estimates for each modeling approach were reported with 95% confidence intervals (CIs). Primary scores from the cognitive, motor, and language domains of the BSID-III were assessed in addition to motor and language subscale scores (fine motor, gross motor; receptive communication, expressive communication). The associations between average prenatal air pollution exposures with developmental outcomes were also examined for non-linearity by fitting generalized additive models (GAMs). Models with statistically significant smooth terms (*p* < 0.05) and effective degrees of freedom greater than one (edf > 1) were flagged as non-linear and investigated based on tertiles of exposure using multivariable linear regression. This included the associations of prenatal PM_10_ exposure with composite, scaled, and gross motor (pGAM < 0.05). For these associations, we also fitted GAMs for each individual monthly exposure during the prenatal period to assess the validity of applying linear DLMs. Among the three outcomes assessed (composite, scaled, and gross motor), each had only one month of PM_10_ exposure that showed some evidence of non-linearity. Therefore, we proceeded to use linear DLMs in all analyses.

All statistical models adjusted for SES, number of breast feedings per day, infant gestational age (early/late/on time), pre-pregnancy BMI (kg/m^2^), infant birthweight (kg) and infant sex. These covariates were chosen by examining choice covariates in relevant literature. However, univariate associations between prenatal exposures and neurodevelopment outcomes are also reported in Supplemental Table 2. Based on previous studies, [14, 16] infant sex was also examined as a potential effect modifier of the relationships between prenatal ambient air pollution and neurodevelopmental outcomes via an interaction term in multivariable linear regression models. Reported values for descriptive statistics are shown as means ± standard deviations for continuous variables and as percentages (%) for categorical variables. Correlations among pollutants and exposure windows were examined using Spearman’s rank tests. Statistical significance for this study was defined as a two-sided *p-value* less than 0.05 for our models. However, we also report *p-value*s that were adjusted for multiple hypothesis testing when examining the associations between the three average prenatal exposures and the 10 neurodevelopmental outcomes using a false discovery rate (FDR) of 10% with the Benjamini-Hochberg (BH) procedure (P_FDR_ < 0.10). All statistical analyses were performed in R (Version 4.0.2) and figures were made in R or PRISM (Version 9.4).

## Results

**Fig. 1:**
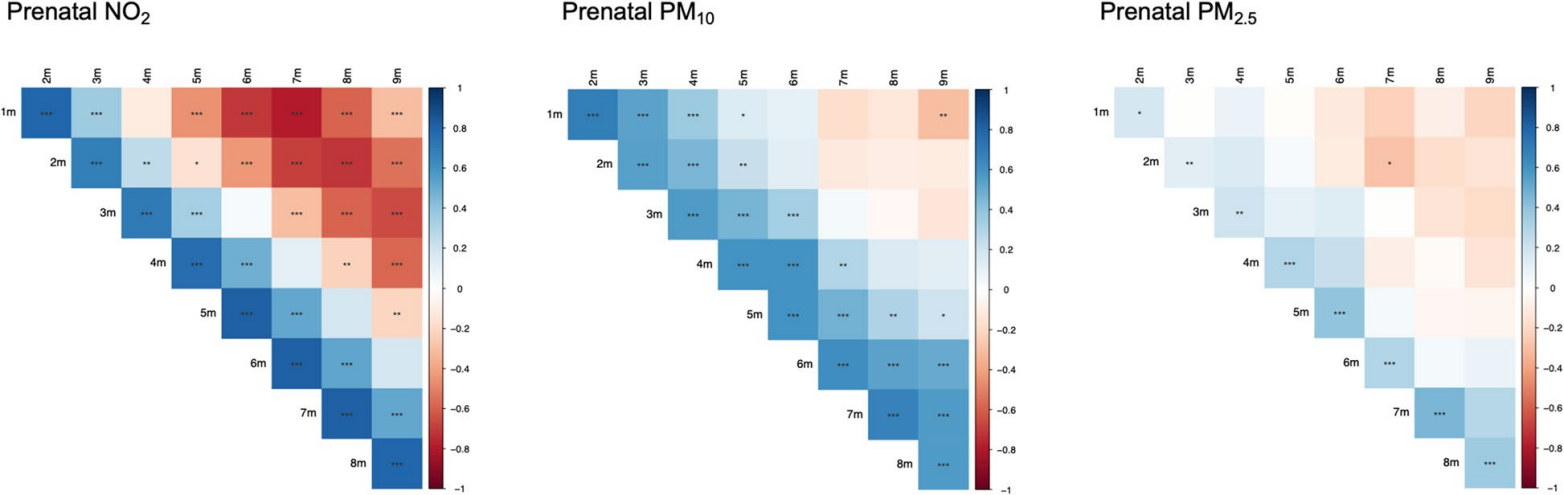
Correlations Among Exposure Windows During the 9-Month Pregnancy Period. Figures show the Spearman correlation structure among individual monthly lags of prenatal ambient air pollution exposure for NO_2_ (left), PM_10_ (middle), and PM_2.5_ (right). Blue colors indicate positive correlations while red colors indicate negative correlations. Statistical significance is denoted as *p < 0.05, **p < 0.01, and ***p < 0.001, respectively

This analysis examined 161 Latino mother-infant pairs (Table 1). Briefly, 57% of infants were female. Average maternal age was 29.02 years (range: 18–45) at enrollment. Mothers in our sample were on average overweight, with an average pre-pregnancy BMI of 28.55 kg/m^2^. Most families were of a low SES according to the Hollingshead index with scores ranging from 3–68 (average: 26.81). Average ambient air pollution exposure for each individual prenatal monthly lag is shown in Table 2. The correlations between average prenatal exposures of PM_10_, PM_2.5_, and NO_2_ ranged from 0.33 to 0.76 (Table 3) while correlations among individual monthly lags of exposure ranged from -0.30 to 0.68 for PM_10_, -0.29 to 0.46 for PM_2.5_, and -0.79 to 0.82 for NO_2_ (Fig. 1). Correlations among individual monthly lags of exposure between pollutants are shown in Supplemental Table 1.

**Table 1:** Characteristics of Mother-Infant Pairs from the Southern California Mother’s Milk Study at the 1-Month Baseline Visit

	**Mean ± SD**
**Maternal Characteristics**	
Maternal Age (years)	29.02 ± 6.21
Pre-pregnancy BMI (kg/m^2^)	28.55 ± 5.82
Maternal BMI (kg/m^2^)	30.16 ± 5.08
Socioeconomic Status	26.81 ± 12.06
**Infant Characteristics**	
Infant Sex F/M (%F)	91/70 (56.5%)
Gestational Age Early/On Time/Late (% On Time)	33/86/42 (53.4%)
Infant Birthweight (kg)	3.40 ± 0.41
Infant Age (days)	32.65 ± 3.99
Breastfeeding / Day	6.73 ± 2.21

Descriptive characteristics of 161 Latino mother-infant pairs from the Southern California Mother’s Milk Study. Data are reported as mean ± standard deviation (SD) unless indicated otherwise. Socioeconomic status (SES) is estimated based off the Hollingshead Four-Factor Index where missing values are replaced by median. Gestational age was based on time of delivery, which included on-time, early (≥ 2 weeks before due date), and late (≥ 2 weeks after due date)

**Table 2:** Monthly Prenatal Average Ambient Air Pollution Exposure Among Mother-Infant Pairs from the Southern California Mothers Milk Study

**Pollutant**	**Means ± SD of Prenatal Exposure Windows**									
	Preg 1 m	Preg 2 m	Preg 3 m	Preg 4 m	Preg 5 m	Preg 6 m	Preg 7 m	Preg 8 m	Preg 9 m	Preg 1-9 m
**NO**_**2**_** (ppb)**	18.17 ± 6.57	18.26 ± 6.12	17.94 ± 5.39	18.22 ± 6.06	18.06 ± 6.67	17.72 ± 6.42	17.54 ± 6.71	17.45 ± 6.62	17.67 ± 6.66	17.89 ± 2.43
**PM**_**10**_** (µg/m**^**3**^**)**	28.37 ± 5.67	29.18 ± 6.24	28.99 ± 5.94	29.38 ± 6.34	30.21 ± 6.79	29.84 ± 6.55	30.29 ± 7.43	30.49 ± 7.30	30.91 ± 6.76	29.74 ± 3.94
**PM**_**2.5**_** (µg /m**^**3**^**)**	11.86 ± 2.60	11.90 ± 2.39	11.50 ± 2.37	11.55 ± 2.64	12.21 ± 3.79	11.83 ± 2.90	11.94 ± 3.56	12.17 ± 3.72	11.93 ± 2.97	11.88 ± 1.24

Means and standard deviations (SD) of ambient air pollutants during each monthly lag of the 9 months pregnancy (Preg) period. As an example, Preg 1 m = 1^st^ month of pregnancy

**Table 3:** Correlations Between Average Prenatal Ambient Air Pollution Exposures

	Prenatal PM_2.5_	Prenatal PM_10_	Prenatal NO_2_
Prenatal PM_2.5_	1		
Prenatal PM_10_	0.76***	1	
Prenatal NO_2_	0.51***	0.33***	1

Table shows the Spearman correlation coefficients between prenatal ambient air pollution exposures. Statistical significance is denoted as ***p < 0.001

### Prenatal exposure to ambient air pollution was associated with neurodevelopmental outcomes at 2 years

**Fig. 2:**
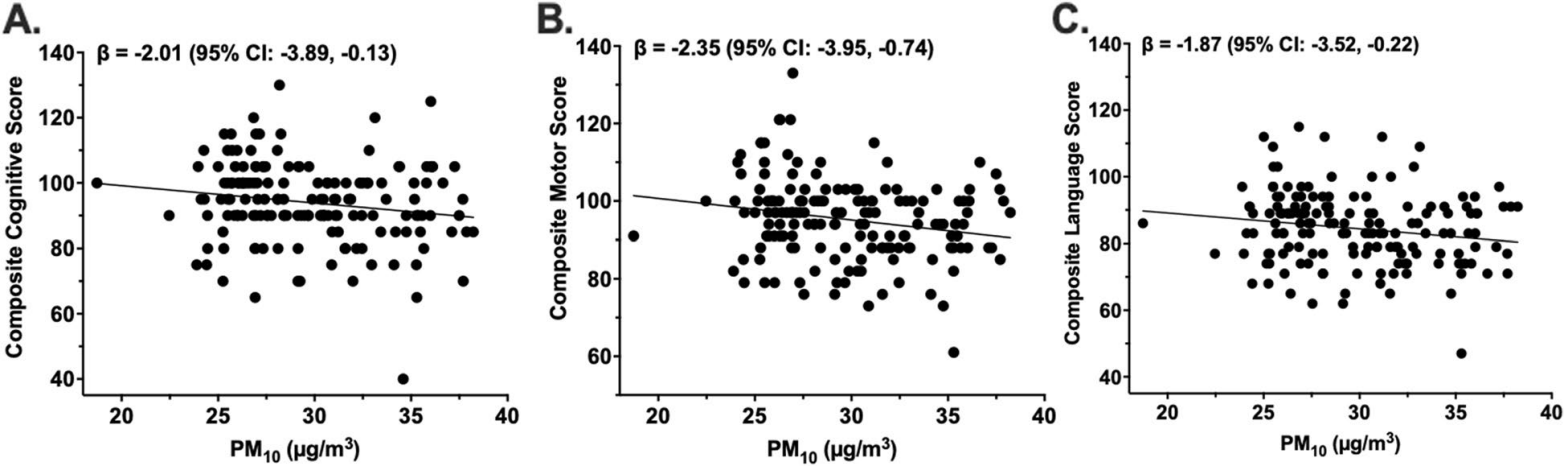
Associations Between Prenatal PM_10_ Exposure and Composite Cognitive, Motor, and Language Scores at 2 Years. Average prenatal exposure to PM_10_ (µg/m^3^) was inversely associated with composite cognitive score (**A**), composite motor score (**B**), and composite language score (**C**). Unadjusted plots and regression lines for neurodevelopmental scores and prenatal PM_10_ are shown. Figures show betas (β) and 95% confidence intervals (CIs) that were scaled to a 1SD difference in exposure (SD NO_2_ = 2.43 ppb, SD PM_10_ = 3.94 µg/m^3^, SD PM_2.5_ = 1.24 µg/m^3^) from multivariable linear regression models that adjusted for socioeconomic status (SES), breast feedings per day, gestational age, pre-pregnancy BMI, infant birthweight, and infant sex

Higher prenatal PM_10_ and PM_2.5_ exposure were associated with lower cognitive outcome scores at 2 years of age (Table 4). Specifically, prenatal exposure to PM_10_ and PM_2.5_ were negatively associated with composite cognitive score (β = -2.01, *p* = 0.04; β = -1.97, *p* = 0.04, respectively). In addition, prenatal PM_10_ exposure was negatively associated with all measures related to motor function, including fine motor scaled score (β = -0.40, *p* = 0.01), gross motor scaled score (β = -0.37, *p* = 0.03), total motor scaled score (β = -0.77, *p* < 0.01), and composite motor score (β = -2.35, *p* < 0.01). Prenatal exposure to PM_10_ was also inversely associated with composite language score (β = -1.87, *p* = 0.03), scaled language score (β = -0.61, *p* = 0.03), and expressive communication score (β = -0.36, *p* = 0.02). Of note, and as shown in Fig. 2, PM_10_ was negatively associated with the composite scores for each of the neurodevelopmental domains examined in this study. Average prenatal exposure to NO_2_ was not associated with neurodevelopmental outcomes at 2 years of age. After correction for multiple hypothesis testing, only prenatal PM_10_ exposure remained significantly associated with motor scaled score (*P*_FDR_ = 0.069) and composite motor score (*P*_FDR_ = 0.069) at a 10% false discovery rate. Results from univariate models were largely consistent except for the association between prenatal PM_2.5_ and composite cognitive score, which was no longer statistically significant when not adjusting for our a priori covariates (Supplemental Table 2). Additionally, only the associations between prenatal NO_2_ with gross motor scaled score and language composite score differed by infant sex (*P*_int_ = 0.02 and *P*_int_ = 0.05, respectively). Specifically, prenatal NO_2_ was positively associated with gross motor scaled score among females (β = 0.20, *p* = 0.03) but not males (β = -0.16, *p* = 0.14). Also, prenatal NO_2_ tended to be negatively associated with gross motor scaled score among males (β = -0.96, *p* = 0.06) but not females (β = 0.62, *p* = 0.20). No other associations between prenatal ambient air pollution exposure and neurodevelopmental outcomes differed by infant sex (P_interactions_ > 0.05). Lastly, GAMs for the associations between prenatal PM_10_ exposure and motor scores demonstrated some evidence of non-linearity (pGAM = 0.007, edf = 3.03; pGAM = 0.04, edf = 2.95 and pGAM = 0.01, edf = 3.0, respectively) (Supplement Fig. 1). To identify the best modeling approach, we ran GAMs containing separate terms for the linear and non-linear effects of PM_10_ and found that the non-linear effects of PM_10_ with composite, scaled, and fine motor scores were statistically significant (*p* = 0.04, *p* = 0.04, *p* = 0.05, respectively), while the linear effects were not. Plots of the fitted associations appeared cubic, therefore we examined prenatal PM_10_ exposure in tertiles (n = 54 and 18.7–27.1 µg/m^3^, n = 53 and 27.1–31.2 µg/m^3^, n = 54 and 31.2–38.2 µg/m^3^) to explore these non-linear associations. Overall, positive, yet non-statistically significant, associations were observed between prenatal PM_10_ exposure with composite, scaled, and fine motor scores in the bottom (β = 1.60, β = 0.57, β = 0.03, respectively) and top tertiles (β = 3.35, β = 1.10, β = 0.58, respectively). Whereas negative associations between prenatal PM_10_ exposure with composite, scaled, and gross motor scores were observed in the middle tertile (β = -6.54, β = -2.12, β = -0.86, respectively).

**Table 4:** Associations Between Average Prenatal Exposure to Ambient Air Pollutants and Neurodevelopmental Outcomes at 2 Years

		**NO**_**2**_			**PM**_**10**_			**PM**_**2.5**_		
		β	95% CI	p	β	95% CI	p	β	95% CI	p
**Motor Scores**	Fine Motor Score	0.05	-0.26, 0.37	0.74	-0.40	-0.71, -0.09	**0.01**	-0.27	-0.58, 0.04	0.09
	Gross Motor Score	0.09	-0.25, 0.43	0.59	-0.37	-0.70, -0.04	**0.03**	-0.13	-0.46, 0.20	0.44
	Motor Scaled Score	0.19	-0.36, 0.73	0.51	-0.77	-1.30, -0.24	0.005*	-0.41	-0.94, 0.13	0.14
	Composite Motor Score	0.55	-1.11, 2.22	0.51	-2.35	-3.95, -0.74	0.004*	-1.22	-2.84, 0.40	0.14
**Cognitive**	Cognitive Scaled Score	-0.06	-0.42, 0.30	0.75	-0.34	-0.70, 0.01	0.06	-0.28	-0.64, 0.07	0.12
	Composite Cognitive Score	-0.70	-2.62, 1.22	0.47	-2.01	-3.89, -0.13	**0.04**	-1.97	-3.83, -0.10	**0.04**
**Language Scores**	Receptive Communication Score	-0.03	-0.37, 0.31	0.87	-0.25	-0.59, 0.08	0.13	-0.18	-0.52, 0.15	0.28
	Expressive Communication Score	-0.07	-0.38, 0.24	0.65	-0.36	-0.66, -0.05	**0.02**	-0.15	-0.46, 0.16	0.34
	Language Scaled Score	-0.10	-0.68, 0.48	0.74	-0.61	-1.18, -0.05	**0.03**	-0.33	-0.90, 0.24	0.25
	Composite Language Score	-0.33	-2.02, 1.36	0.70	-1.87	-3.52 -0.22	**0.03**	-1.01	-2.67, 0.65	0.23

Table shows the results from multivariable linear regression analyses between prenatal ambient air pollution exposure and cognitive outcomes assessed at 2 years in Latino infants. Models adjusted for socioeconomic status (SES), breast feedings per day, gestational age, pre-pregnancy BMI, infant birthweight, and infant sex. Table shows betas (β) and 95% confidence intervals (CIs) that were scaled to a 1SD difference in exposure (SD NO_2_ = 2.43 ppb, SD PM_10_ = 3.94 µg/m^3^, SD PM_2.5_ = 1.24 µg/m^3^). Statistical significance is denoted in bold (*p* < 0.05) while * and ** represent an adjusted *p-value* of *p* < 0.10 and *p* < 0.05 respectively

### Mid/Late prenatal ambient air pollution was inversely associated with neurodevelopmental outcomes at 2 years

**Fig. 3:**
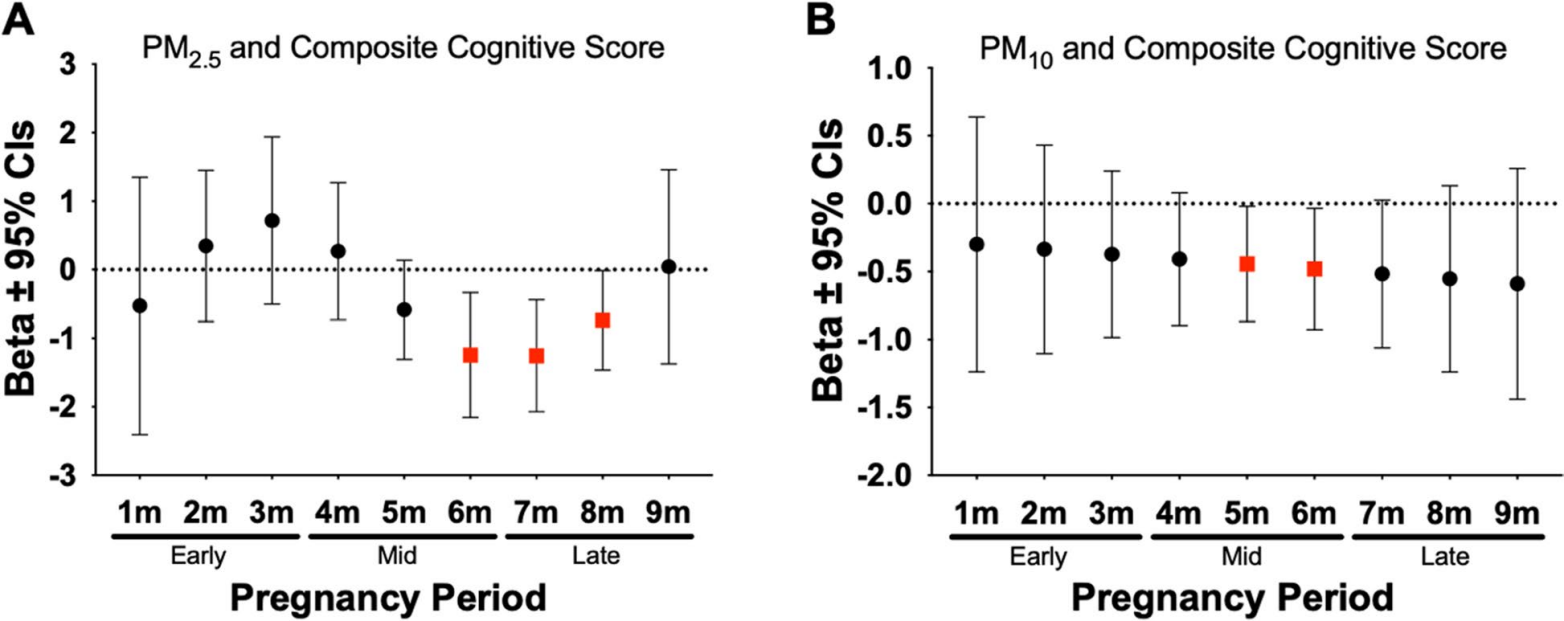
Associations Between PM_10_ and PM_2.5_ Exposure During Mid to Late Pregnancy and Composite Cognitive Scores at 2 Years. Figures show effect sizes and 95% confidence intervals (CI) at each monthly lag of exposure during the pregnancy period. Results were obtained from distributed lag models (DLMs) that adjusted for socioeconomic status (SES), breast feedings per day, gestational age, pre-pregnancy BMI, infant birthweight, and infant sex. Panels show associations between PM_2.5_ and composite cognitive score (**A**), PM_10_ and composite cognitive score (**B**). Effect sizes are scaled by the IQR of each respective pollutant (PM_10_ = 8 µg/m^3^, PM_2.5_ = 3 µg/m^3^). Statistically significant windows are denoted by red squares (*p* < 0.05)

**Fig. 4:**
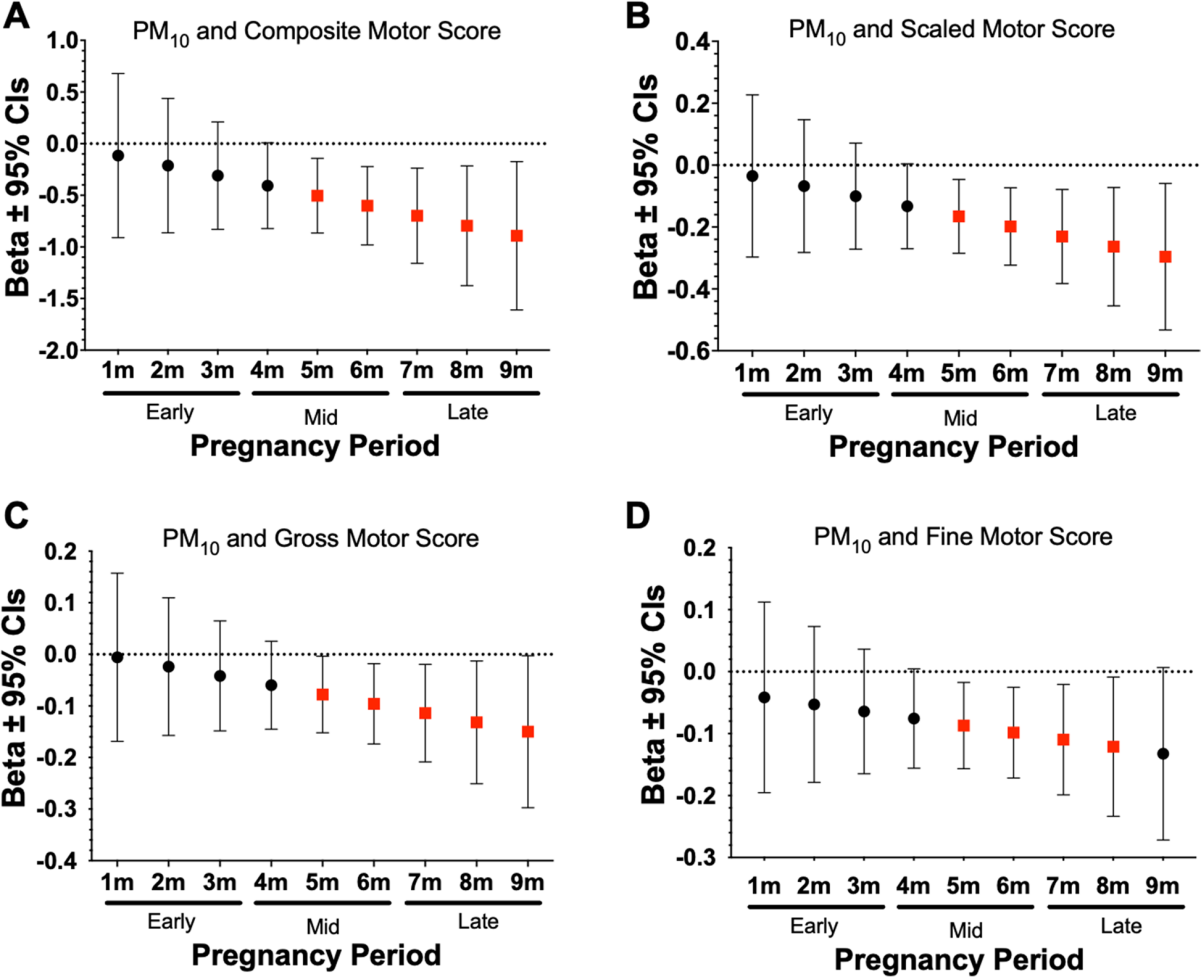
Associations Between PM_10_ Exposure During Mid and Late Pregnancy and Motor Scores at 2 Years. Figures show effect sizes and 95% confidence intervals (CI) at each monthly lag of exposure during the pregnancy period. Results were obtained from distributed lag models (DLMs) that adjusted for socio-economic status, breast feedings per day, gestational age, pre-pregnancy BMI, infant birthweight, and infant sex. Panels show associations between PM_10_ and composite motor score (**A**), scaled motor score (**B**), gross motor score (**C**) and fine motor score (**D**). Effect sizes are scaled by the IQR (PM_10_ = 8 µg/m^3^). Statistically significant windows are shown in red (*p* < 0.05)

**Fig. 5:**
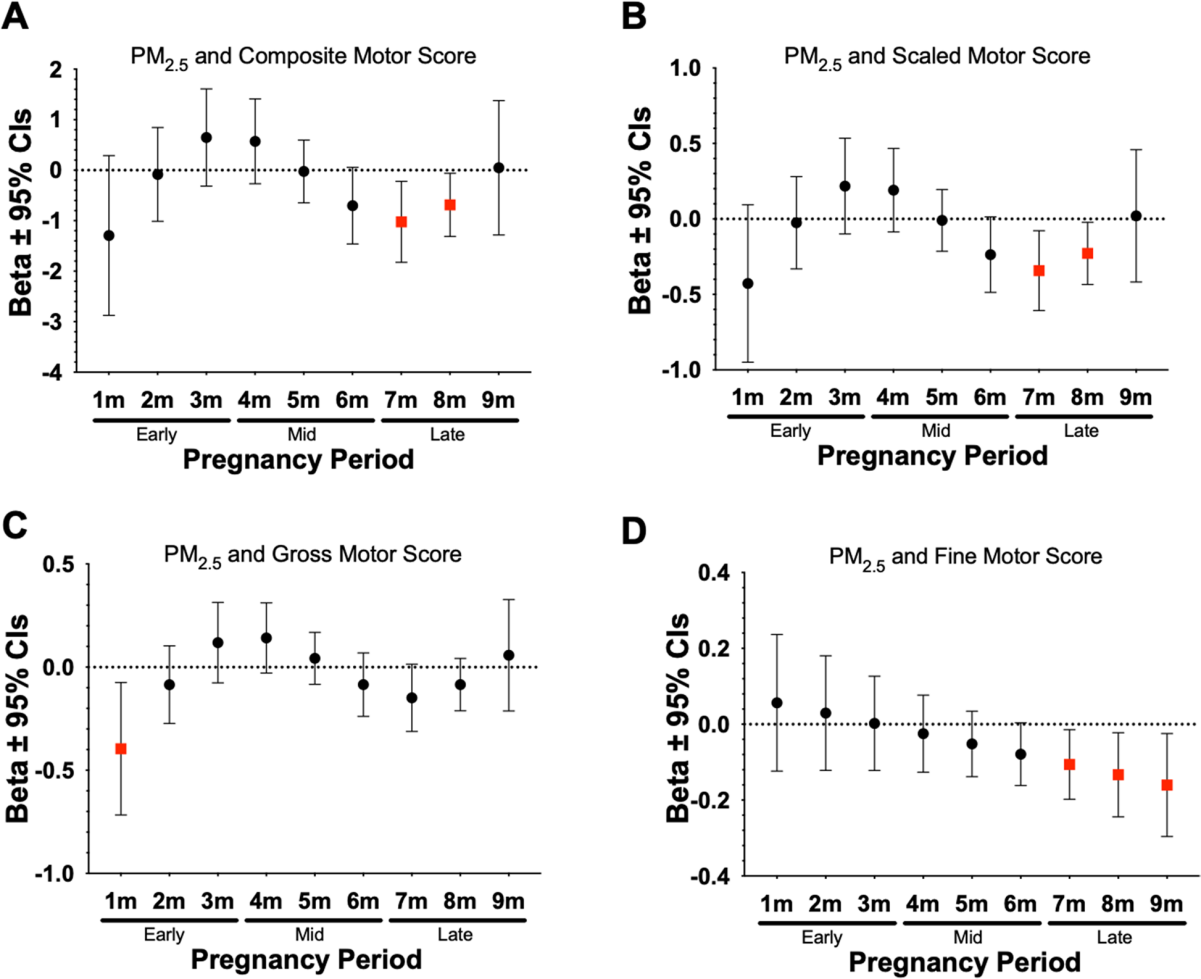
Associations between PM_2.5_ Exposure During Mid to Late Pregnancy and Motor Scores at 2 Years. Figures show effect sizes and 95% confidence intervals (CI) at each monthly lag of exposure during the pregnancy period. Results were obtained from distributed lag models (DLMs) that adjusted for socio-economic status, breast feedings per day, gestational age, pre-pregnancy BMI, infant birthweight, and infant sex. Panels show associations between PM_2.5_ and composite motor score (**A**), scaled motor score (**B**), and fine motor score (**C**). Effect sizes are scaled by the IQR (PM_2.5_ = 3 µg/m^3^). Statistically significant windows are shown in red (*p* < 0.05)

DLMs were used to examine monthly prenatal ambient air pollution exposures to identify critical windows of exposure that may be related to neurodevelopmental outcomes at 2 years of age. Results from this analysis indicated that prenatal exposure during the 1 to 5 months prior to birth was associated with neurodevelopmental outcomes at 2 years of age. For instance, we observed significant negative associations between prenatal exposure to PM_10_ and PM_2.5_ and both composite and scaled cognitive scores during mid/late pregnancy (Fig. 3). As shown in Fig. 4, significant negative associations were also found between prenatal PM_10_ and composite, scaled, gross, and fine motor scores during mid/late pregnancy. In addition, higher prenatal PM_2.5_ during late pregnancy was significantly associated with lower composite, scaled, and fine motor scores during late pregnancy (Fig. 5). Lastly, as shown in Table 5 we observed several other significant negative associations between NO_2_, PM_10_, and PM_2.5_ during specific prenatal months and other neurodevelopmental scores. Results from all the DLM models can be found in Supplemental Table 3.

**Table 5:** Associations Between Specific Windows of Prenatal Ambient Air Pollution Exposure and Neurodevelopmental Outcomes at 2 Years

Outcome	Beta	95% CI
**Gross Motor Score**		
NO_2_ 1 m (early pregnancy)	-0.35	(-0.69, -0.002)
**Composite Cognitive Score**		
NO_2_ 9 m (late pregnancy)	-1.76	(-3.50, -0.03)
**Composite Language Score**		
PM_2.5_ 6 m (mid pregnancy)	-0.84	(-1.58, -0.09)
PM_2.5_ 7 m (late pregnancy)	-1.25	(-2.09, -0.40)
PM_2.5_ 8 m (late pregnancy)	-0.73	(-1.37, -0.08)
PM_10_ 5 m (mid pregnancy)	-0.41	(-0.78, -0.03)
PM_10_ 6 m (mid pregnancy)	-0.47	(-0.86, -0.08)
PM_10_ 7 m (mid pregnancy)	-0.53	(-1.01, -0.05)
NO_2_ 8 m (late pregnancy)	-1.15	(-2.23, -0.06)
**Scaled Language Score**		
PM_2.5_ 6 m (mid pregnancy)	-0.28	(-0.53, -0.03)
PM_2.5_ 7 m (late pregnancy)	-0.42	(-0.71, -0.13)
PM_2.5_ 8 m (late pregnancy)	-0.25	(-0.47, -0.02)
PM_10_ 5 m (mid pregnancy)	-0.13	(-0.26, -0.01)
PM_10_ 6 m (mid pregnancy)	-0.15	(-0.29, -0.02)
PM_10_ 7 m (late pregnancy)	-0.17	(-0.34, -0.01)
NO_2_ 1 m (early pregnancy	-0.97	(-1.91, -0.03)
NO_2_ 8 m (late pregnancy)	-0.38	(-0.75, -0.01)
**Expressive Communication Score**		
PM_2.5_ 7 m (late pregnancy)	-0.16	(-0.30, -0.01)
PM_10_ 4 m (mid pregnancy)	-0.09	(-0.17, -0.01)
PM_10_ 5 m (mid pregnancy)	-0.08	(-0.15, -0.01)
PM_10_ 6 m (mid pregnancy)	-0.08	(-0.15, -0.004)
**Receptive Communication Score**		
PM_2.5_ 7 m (late pregnancy)	-0.11	(-0.20, -0.01)
PM_2.5_ 8 m (late pregnancy)	-0.15	(-0.27, -0.03)
PM_2.5_ 9 m (late pregnancy)	-0.20	(-0.34, -0.05)
PM_10_ 7 m (late pregnancy)	-0.10	(-0.20, -0.01)
PM_10_ 8 m (late pregnancy)	-0.13	(-0.25, -0.01)
PM_10_ 9 m (late pregnancy)	-0.15	(-0.31, -0.003)
NO_2_ 8 m (late pregnancy)	-0.22	(-0.43, -0.01)
NO_2_ 9 m (late pregnancy)	-0.36	(-0.67, -0.06)

Table shows the significant associations between monthly ambient air pollution exposure during pregnancy and cognitive outcomes at 2 years of age where non-statistically significant findings are not shown. Effect estimates and 95% confidence intervals (CI) were obtained from distributed lag models (DLMs) that adjusted for socioeconomic status (SES), breast feedings per day, gestational age, pre-pregnancy BMI, infant birthweight, and infant sex. Effect estimates are scaled to an IQR increase in exposure (IQR PM_2.5_ = 3 µg/m^3^, IQR PM_10_ = 8 µg/m^3^, IQR NO_2_ = 9 ppb)

## Discussion

Our analysis revealed that higher average prenatal exposure to PM_10_ and PM_2.5_ was adversely associated with functional neurodevelopmental outcomes at 2 years. As an example, those exposed to PM_10_ levels at the 75^th^ percentile (32.50 µg/m^3^) compared to the 25^th^ percentile (26.64 µg/m^3^) of exposure were predicted to have a three point lower composite cognitive score. For context, in the current study, 16% of participants had a composite cognitive score that indicated some degree of impairment [40, 41]. If all participants had PM_10_ levels at the 75^th^ percentile of exposure, the prevalence of cognitive impairment would be predicted to increase to 22%, which highlights the importance of even moderate increases in early-life exposure. Lastly, distributed lag modeling suggested that exposures during mid/late were inversely associated with neurodevelopment. These exposure periods overlap with developmental processes such as myelination, neuronal migration, synaptogenesis, apoptosis, and rapid neurogenesis and circuit formation that support the emergence of functional sensory systems, motor systems, and connectivity networks [23–26]. These findings support the growing body of work that indicates adverse effects of prenatal air pollution exposure on cognitive development and further suggest the presence of critical neurodevelopmental windows which exhibit increased sensitivity to environmental exposures. [10–20]

While the exact mechanisms by which prenatal ambient air pollution may impact neurodevelopment have yet to be fully characterized, current evidence suggests that these exposures can increase neuroinflammation and oxidative stress that may interfere with brain development. In utero, air pollutants may come into direct contact with the fetus where black carbon has been found on the fetal side of the placenta, [42] and rodent studies have found nanoparticles in fetal blood following prenatal exposure to diesel exhaust [43]. Further, exposure to ambient air pollution has been associated with increased activation of astroglia and microglia *in vitro* [44] that may negatively impact synaptic pruning [45, 46]. PM exposure has also been associated with increased levels of inflammatory mediators in brain tissue, cerebrospinal fluid, and serum from individuals with chronic exposure to air pollution [47–50]. Lastly, while there is mixed evidence regarding whether pro-inflammatory cytokines can successfully cross the placenta, [51–53] it is well established that the placenta is capable of its own cytokine production [54–57] where acute and chronic maternal inflammation during pregnancy has been associated with neurodevelopmental disorders [58–62]. This is important since increased levels of neuroinflammation have been linked to morphological changes in the brain in animal models [63, 64]. Together, these studies suggest several mechanisms by which prenatal ambient air pollution may impact neurodevelopment in early life.

While this study adds to a growing body of evidence that prenatal ambient air pollution exposure is associated with neurodevelopmental outcomes in early life, these results should be interpreted in the context of the study’s limitations. Upon adjustment for multiple hypothesis testing, only the associations between PM_10_ and composite and scaled motor score remained statistically significant as determined by a 10% false discovery rate. However, this may be due to our relatively small sample since many of the associations that we observed in the current study are consistent with other work [10, 11, 16, 65]. Further, our small sample size limits our ability to draw conclusions regarding single sex-specific associations as well as the interpretability of our non-linear models. Since this study was restricted to Latino infants, generalizability of our results may be limited; however, Latino communities are known to experience higher disease burden, disproportionate levels of air pollution exposure that may adversely impact human health, and remain understudied in biomedical research [2–4, 66–68]. For example, average 9-month prenatal exposure levels observed in our cohort were higher than the most recent World Health Organization guidelines for yearly air quality where average PM_2.5_, PM_10_, and NO_2_ exposures were approximately 6.88 µg/m^3^, 14.74 µg/m^3^, and 12 ppb higher than current guidelines, respectively. [1]

In the current study, neurodevelopmental assessments were made using the BSID-III, which is one of the most commonly used assessments for infant development [69, 70]. Nevertheless, the accuracy of the BSID-III in predicting future outcomes is limited; BSID-III cognitive scores at 2 years, for example, have been found to overestimate performance when compared with full-scale IQ at 4 years. [71] Also, while we did not have data regarding home environment or maternal IQ, all models adjusted for SES in an attempt to capture some of these potentially important factors in child neurodevelopment [72, 73]. Additionally, since prenatal pollution estimates were based on geocoded residential addresses, they may not fully reflect maternal time-activity patterns and do not capture indoor sources of air pollution. While these factors may contribute to exposure misclassification, this would be random amongst the sample and would likely bias results to the null [74]. Further, since the current study focused on prenatal exposures, we cannot rule out the potential importance of postnatal exposures and their impact on neurodevelopment. Future work in this cohort will aim to expand these analyses by examining the relative importance of pre- and postnatal exposures on neurodevelopment. Lastly, while this study looked at several air pollutants, we were unable to assess multipollutant models during pregnancy due to the correlation among NO_2_, PM_10_, and PM_2.5_ exposure. However, by using DLMs for each pollutant, we were able to identify specific time windows of exposure that appear to be important for neurodevelopment in infants.

## Conclusion

Prenatal exposure to ambient air pollutants was inversely associated with functional neurodevelopmental outcomes at 2 years, raising concern for child health and future functional impairment. In addition, our findings indicate that exposures during mid to late pregnancy may be especially detrimental to neurodevelopment, which suggests the need for limiting air pollution exposure, especially during the latter half of pregnancy. In summary, this study adds to the growing body of literature cataloging the negative health consequences of both pre- and postnatal ambient air pollution exposure that should be used to inform policy efforts to limit human exposure to air pollutants.

## Supplementary Information

**Additional file 1: Supplemental Figure 1.** Average Prenatal PM_10_ Exposure Demonstrated Non-Linear Associations with Composite, Fine, and Scaled Motor Scores at 2 Years.

**Additional file 2: Supplemental Table 1.** Correlations of Monthly Exposure Lags Between Ambient Air Pollutants During the 9-Month Pregnancy Period.

**Additional file 3: Supplemental Table 2.** Univariate Associations Between Average Prenatal Exposure to Ambient Air Pollutants and Neurodevelopmental Outcomes at 2 Years.

**Additional file 4: Supplemental Table 3.** Associations Between All Windows of Prenatal Ambient Air Pollution Exposure and Neurodevelopmental Outcomes at 2 years.

## Data Availability

The datasets used and/or analyzed during the current study are available from the corresponding author on reasonable request.
