# Supplementary material for: Prenatal exposure to ambient air pollution is associated with neurodevelopmental outcomes at 2 years of age
Source: Environ Health. 2023 Jan 24;22:11. doi: 10.1186/s12940-022-00951-y (PMC9872424; doi:10.1186/s12940-022-00951-y)
Supplement: Supplementary file 1 — Additional file 1: Supplemental Figure 1. Average Prenatal PM10 Exposure Demonstrated Non-Linear Associations with Composite, Fine, and Scaled Motor Scores at 2 Years. [file 12940_2022_951_MOESM1_ESM.docx]

**Supplemental Figure 1.** Average Prenatal PM_10_ Exposure Demonstrated Non-Linear Associations with Composite, Fine, and Scaled Motor Scores at 2 Years


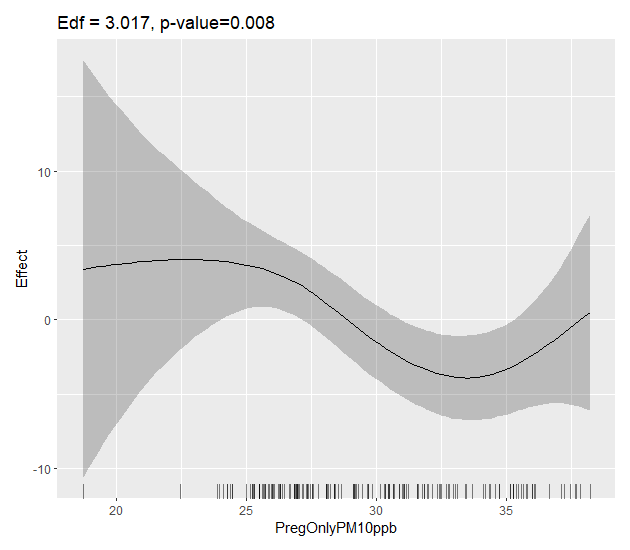

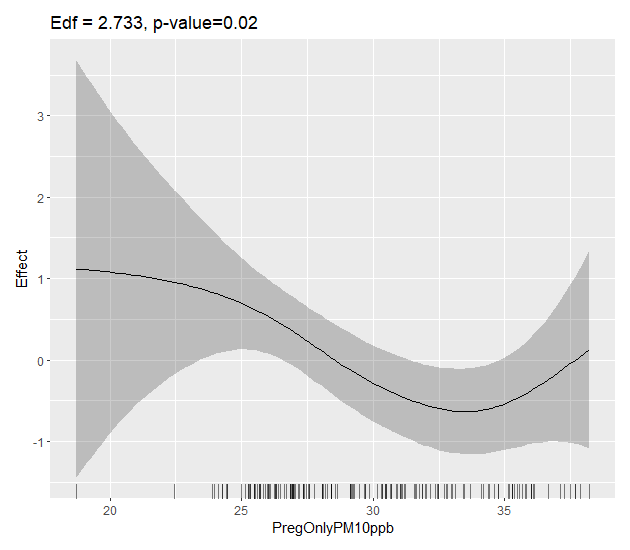

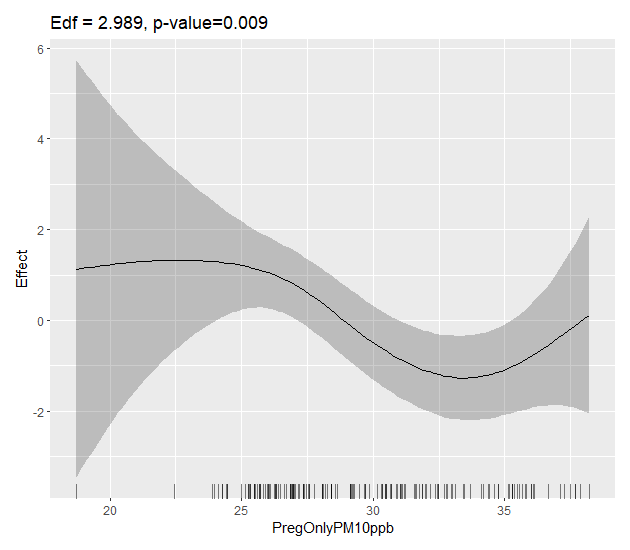


A

B

C

Prenatal PM_10_

**Composite Motor**

Prenatal PM_10_

**Fine Motor**

Prenatal PM_10_

**Scaled Motor**

Effect

Effect

Effect

Edf = 3.017, p-value = 0.008

Edf = 2.733, p-value = 0.02

Edf = 2.989, p-value = 0.009

**Supplemental Figure 1.** Figures show effect sizes for non-linear associations between average prenatal PM_10_ and composite, fine, and scaled motor score at 2 years. Results were obtained from generalized additive models (GAMs) that adjusted for socio-economic status (SES), breast feedings per day, gestational age, pre-pregnancy BMI, infant birthweight, and infant sex. Panels show relationship between average prenatal PM_10_ and composite motor score (A), fine motor score (B), and scaled motor score (C). Effective degrees of freedom and exact p-values are reported on each panel, but each model was statistically significant p<0.05.
